# Acute Lung Injury Complicating Blood Transfusion in Post-Partum Hemorrhage: Incidence and Risk Factors

**DOI:** 10.4084/MJHID.2014.069

**Published:** 2014-10-22

**Authors:** Luciana Teofili, Maria Bianchi, Bruno A. Zanfini, Stefano Catarci, Rossella Sicuranza, Serena Spartano, Gina Zini, Gaetano Draisci

**Affiliations:** 1 Hematology Department, Università Cattolica del Sacro Cuore, Rome, Italy; 2 Anesthesiology Department, Università Cattolica del Sacro Cuore, Rome, Italy

## Abstract

**Background:**

We retrospectively investigated the incidence and risk factors for transfusion-related acute lung injury (TRALI) among patients transfused for post-partum hemorrhage (PPH).

**Methods:**

We identified a series of 71 consecutive patients with PPH requiring the urgent transfusion of three or more red blood cell (RBC) units, with or without transfusion of fresh frozen plasma (FFP) and/or platelets (PLT). Clinical records were then retrieved and examined for respiratory distress events. According to the 2004 consensus definition, cases of new-onset hypoxemia, within 6 hours after transfusion, with bilateral pulmonary changes, in the absence of cardiogenic pulmonary edema were identified as TRALI. If an alternative risk factor for acute lung injury was present, possible TRALI was diagnosed.

**Results:**

Thirteen cases of TRALI and 1 case of possible TRALI were identified (overall incidence 19.7%). At univariate analysis, patients with TRALI received higher number of RBC, PLT and FFP units and had a longer postpartum hospitalization. Among the diseases occurring in pregnancy- and various pre-existing comorbidities, only gestational hypertension and pre-eclampsia, significantly increased the risk to develop TRALI (p = 0.006). At multivariate analysis including both transfusion- and patient-related risk factors, pregnancy-related, hypertensive disorders were confirmed to be the only predictors for TRALI, with an odds ratio of 27.7 ( 95% CI 1.27–604.3, p=0.034).

**Conclusions:**

Patients suffering from PPH represent a high-risk population for TRALI. The patients with gestational hypertension and pre-eclampsia, not receiving anti-hypertensive therapy, have the highest risk. Therefore, a careful monitoring of these patients after transfusions is recommended.

## Introduction

Postpartum hemorrhage (PPH) constitutes the most frequent cause of maternal mortality in low-income countries.^1^ In developed countries, PPH is the prevalent cause of critical illness among obstetric patients, and recent evidences suggest that its incidence is progressively rising.^2^,^3^ In particular, women with persistent PPH, defined as “the active bleeding exceeding 1000 ml within 24 hours following delivery, that continues despite the use of initial measures including first-line uterotonic agents and uterine massage”, are considered at high risk of adverse outcome.^4^ Transfusion-related acute lung injury (TRALI) is a serious transfusion reaction characterized by non-cardiogenic lung oedema, hypoxemia and respiratory distress occurring after blood transfusion.^5^–^7^ The reported incidence of TRALI greatly differs in retrospective and prospective studies: overall, it is estimated to vary between 0.08% and 15% of patients receiving a blood transfusion.^5^ According to the two-hit hypothesis, TRALI results from a capillary leak caused by two consecutive events: the adhesion of primed neutrophils to pulmonary endothelial cells (first hit) and, the subsequent activation of both cells by antibodies or inflammatory mediators present in transfused blood (second hit).^8^ Antibodies to class II- human leukocyte antigens and to human neutrophil antigens, contained in donations from persons with a history of transfusions or previous pregnancies, are often implicated in the antibody-mediated TRALI 8. Moreover, various substances accumulated during the prolonged storage of RBC or PLT are suspected to elicit antibody-negative TRALI.^8^ TRALI is particularly observed in critically ill patients: massive transfusion, mechanical ventilation, sepsis, hematological malignancies, end stage liver disease and cardiac surgery are all acknowledged important risk factors for TRALI.^9^–^15^ Since patients with PPH receive transfusion of great amounts of blood products, it is conceivable that they might be at high risk for developing TRALI. Therefore, we retrospectively identified a series of patients heavily transfused for PPH, and we evaluated among them the incidence and risk factors for TRALI.

## Patients and Methods

### Study design

This study is retrospective and was conducted at a tertiary care medical center (Policlinico “A. Gemelli”, Rome). The study included patients with PPH consecutively admitted to the delivery room of our hospital from January 2005 to December 2011, receiving at least three units of red blood cell (RBC) within 24 hours after delivery. TRALI was defined according to 2004 consensus criteria 6,7. The study was approved from the institutional Ethics Board.

### Clinical data collection and TRALI definition

We identified in the blood bank transfusion database EmoNet (Insiel, Trieste, Italy) the patients needing an urgent transfusion of a minimum of three RBC units in the delivery room, with or without fresh frozen plasma (FFP) and platelet concentrates (PLT). Clinical records of patients were then retrieved and, for each patient, were collected demographics, detailed clinical and obstetric history, laboratory and radiological findings and data on administered drugs. In order to identify cases of TRALI, two anesthesiologists (B.A.Z. and S.C.) independently examined clinical records. Cases, requiring arterial blood gas analysis and special oxygen therapy or ventilatory support, were carefully checked; clinical information was then matched with transfusion history. According to the 2004 consensus criteria, TRALI was diagnosed in the event of new-onset hypoxemia or deterioration demonstrated by a PaO2/FiO2 <300 mmHg within 6 hours after transfusion, with bilateral pulmonary changes in the absence of cardiogenic pulmonary edema.^6^ If an alternative risk factor for acute lung injury (ALI) was present, the diagnosis of possible TRALI (pTRALI) was performed.^6^,^7^ Patients were then grouped according to the outcome “TRALI” or “no TRALI”. The following variables were compared between groups: age, smoking, presence of disseminated intravascular coagulopathy – DIC, fibrinogen and hemoglobin prepartum levels, number of previous pregnancies, parity, vaginal or cesarean section delivery, need for surgical repair of the uterus (including uterine balloon tamponade, uterine compression sutures, uterine artery ligation and peripartum hysterectomy), presence of morbidities preexisting to pregnancy, pregnancy-related non-hypertensive and hypertensive disorders, post-delivery hospitalization time, rate of admission at intensive care unit (ICU) and estimated blood loss.

### Transfusion data collection

Blood products were supplied from the blood bank of Policlinico “A. Gemelli”; blood product data were collected using the EmoNet database (Insiel). We evaluated the following transfusion-related risk factors for TRALI: number of units received of RBC, PLT and FFP, number of units received from female donors, mean storage time of transfused units (for RBC and PLT), number of RBC units with a storage time longer than 14 days. All RBC units were in saline, adenine, glucose and mannitol (SAGM) additive solution and were both leukoreduced and buffy-coat removed; PLT concentrates were obtained from buffy-coats of single donors or from apheresis; similarly, FFP units were from both single donors or from apheresis. For the purpose of analysis, we calculated one PLT apheresis unit as five single donor units and one FFP apheresis unit as two single donor units.

## Statistics

Continuous normally distributed variables are expressed as means and standard deviation (SD) and not normally distributed variables as medians (interquartile ranges, IQR). Normality distribution was evaluated through the Shapiro-Wilk normality test. Categorical variables are expressed as n, (%). We first examined at univariate analyses the effect on the outcome (TRALI, no TRALI) of patient-related factors and blood product characteristics. For continuous variables, we used the Mann-Whitney U test or Student’s t test, as appropriate; for categorical variables we used the Fisher’s exact tests. Statistical significance was defined as p ≤ 0.05. Multiple logistic regression using the Enter method 16 was applied to the outcomes: factors achieving p < 0.10 in univariate analyses were entered into the models predicting the risk for the onset of TRALI. Results were expressed as odds ratio (OR) with relative 95% confidence interval (95% CI). Analyses were performed using the IBM SPSS Statistics 21.0 software.

## Results

Among the 22,344 deliveries occurred at our hospital from January 2005 to December 2011, we identified 71 patients with PPH requiring the transfusion of at least three RBC units. The mean age ± SD was 34 ± 5.5 years; 21% of patients had a vaginal delivery and 79% cesarean delivery. The reported estimated blood loss varied from 300 to 7000 ml.

### TRALI diagnosis

We found evidence of a new-onset hypoxemia within 6 hours after transfusion in 15 cases: TRALI were identified in 10 of them, possible TRALI (4 cases) was diagnosed in one patient with pneumonia and in three patients with pre-eclampsia, whilst in one patient with pre-existing valvular heart disease, hypoxemia and pulmonary edema were attributed to transfusion-associated circulatory overload (TACO).^17^ Five patients in the TRALI group (36%) and 5 patients in the no-TRALI group (9%) required admission to ICU (*p=0.021*, Table 2); overall, no patient required ventilatory support for more than 96 hours and no patient died. TRALI cases were not notified to the local hemovigilance office; no immunologic studies in patient and donor samples were performed.

### Transfusion-related risk factors

Among 71 patients with PPH, 20 (28% ) were transfused with only RBC, 44 (62%) with RBC and FFP and 7 (10%) with RBC, FFP and PLT. Overall, the number of patients receiving all the three blood products was higher in the TRALI than in no-TRALI group (*p=0,025*, Table 1). As shown in Table 1, patients with TRALI received a higher number of units of RBC (*p=0,008*), PLT (*p =0,008*) and FFP (*p =0,034*). Overall, patients in the TRALI group received a higher number of blood products from female donors (*p =0,047,* Table 1). RBC and PLT units transfused to patient with or without TRALI had similar storage times and patients in the TRALI group did not receive higher number of “old” RBC units (i.e. those units stored for more than 14 days) (Table 1).

### Patient-related risk factors

The clinical features of patients grouped according to the diagnosis of TRALI are shown in Table 2. TRALI was not associated to age or smoking. Pre-existing diseases were recorded in21 (30 %) patients and included: hypothyroidism (5), obesity (4), heart valvular diseases (2), HCV hepatitis (2), asthma (1), HIV infection (1), Poland syndrome (1 pt), sickle cell disease (1), Marfan syndrome (1 pt), hyperthyroidism (1), type I (1) and type II diabetes mellitus(1), HBV hepatitis (1), liver failure due to Crigler-Najjar disease (1), systemic lupus erythematosus (1); 3 patients had more than one disease. Among patients with pre-existing diseases, seven experienced TRALI (50%) and 14 did not (24%) (*p=0.099*, Table 2). TRALI did not occur in the two patients with heart valvular disease, whereas one of them had TACO. Non-hypertensive co-morbidities related to pregnancy occurred in 7 patients and included anemia (2), gestational diabetes (2), hypothyroidism (2) and intra-hepatic cholestasis (1), in similar proportions between TRALI and no-TRALI groups (Table 2). Eight patients had pregnancy-related hypertensive disorders: 4 suffered from gestational hypertension, 2 were affected by gestational hypertension with superimposed pre-eclampsia, and two patients had pre-eclampsia.^18^ Overall, 5 of them experienced TRALI (Table 2). Indeed, hypertensive disorders were overrepresented among patients with TRALI/possible TRALI (36% versus 5%, *p=0.006*, Table 2). In contrast, patients with and without TRALI had similar obstetric issues, including parity, vaginal or cesarean section delivery, number of previous pregnancies, estimated blood losses and necessity of surgical intervention (Table 2). In addition, other acknowledged risk factors for PPH such as uterine atony, previous uterine surgery, oxytocin administration or placental abnormalities were equally represented among patients with or without TRALI (data not shown). Finally, patients with TRALI were more frequently admitted to the ICU and had a longer hospitalization (*p=0.021* and *p<0.0001*, respectively, Table 2).

### Analysis of combined risk factors

We next examined the concomitant effect of transfusion- and patient-related factors on the risk to develop TRALI, by combining in a multivariate model all factors that in univariate analysis had a significance level of 10% (*p < 0.1*, Table 3). We found that only pregnancy-related hypertensive disorders were significant predictors for TRALI, with an OR of 27.7 (95% CI 1.27–604.3*, p=0.034*) (Table 3). In order to ascertain if these underlying conditions were not merely increasing the risk of transfusion rather than risk of TRALI, we compared the amount of RBC, FFP and PLT transfused to patients with and without hypertensive disorders. We found that patients with hypertensive disorders required similar amounts of blood products as patients without hypertensive disorders (*p=0.699* for RBC, *p=0.685* for FFP and *p=0.325* for PLT, respectively), suggesting that the higher risk for TRALI observed in these patients is not dependent on blood volume transfused.

## Discussion

Previous hemovigilance studies suggest that 6.7 to 15% of reported TRALI occur among obstetrics-gynecological patients,^19^,^20^ but data so far published within in this setting are scarce or even anecdotal.^21^,^22^ In our retrospective series of heavily transfused patients, we found an overall incidence of TRALI/pTRALI of 16,9%, that is noteworthy. We investigated a limited number of patients, representing a fraction of the overall population with PPH admitted to our hospital in the same period. Nonetheless, our study is the first attempt to identify TRALI incidence and risk factors in the obstetrics-gynecology population. In general, critically ill patients have the highest risk to develop TRALI, with reported incidence ranging from 1,8 to 15%.^5^ In PPH patients, as well as in general people, the amounts of transfused products constitute a predictable risk factor for TRALI, with a significant increase of risk in patients receiving all three types of blood components. In order to reduce the TRALI risk, female donors with previous pregnancies or miscarriages as well as donors who had been previously transfused, are currently no longer eligible for plasma donations in many countries.^19^,^23^ Since this policy started in Italy only in mid-2011, many patients included in this study had probably received antibody-positive blood products. Accordingly, also in our series we found a possible implication of blood products from female donors in inducing TRALI. In addition, our findings suggest that in the obstetric setting the predisposition to TRALI is also driven by clinical condition of patients. We found that pregnancy-related hypertensive disorders, encompassing gestational hypertension and pre-eclampsia, are the most significant predictors for TRALI, with an increase of risk for developing this complication of about 27 folds. Severe hypertension with pulmonary edema can frequently complicate preeclampsia;^18^,^24^,^25^, therefore, in pre-eclamptic patients, a careful differential diagnosis with this condition is mandatory. In particular, among our patients with pre-eclampsia, three developed the hypoxemia. In these patients, hypoxemia was not associated with severe hypertension and was not responsive to diuretic administration, suggesting that an underlying cause different from pulmonary edema. Several recent studies demonstrated that various cell types can act as multipliers or attenuators of TRALI, including platelets,^26^ monocytes and T lymphocytes,^27^ and endothelial cells themselves.^28^ Clinical manifestations of pregnancy-related hypertensive disorders reflect a widespread endothelial cell dysfunction, likely due to the release of soluble factors from the ischemic placenta.^24^,^25^ Recently, Caudrillier et al. reported that targeting platelet activation with either aspirin or a glycoprotein IIb/IIIa inhibitor, is also able to protects mice in an experimental TRALI model.^26^ Therefore, even though our multivariate analysis relies on a low number of TRALI patients, the mechanisms underlying pregnancy-related hypertensive disorders support our findings. In fact, a hypothetical platelet-mediated mechanism could underlie pulmonary endothelial breakdown in TRALI and organ vascular damage in pre-eclampsia. Notably, none among our hypertensive patients developing TRALI assumed antiplatelet agents at the time of delivery. Moreover, TRALI occurred in all the three hypertensive patients who did not receive anti-hypertensive therapy but only in two out of five patients receiving alpha-methyldopa, thus suggesting that a tight control of hypertension may help to mitigate the TRALI risk.

The main limitation of our study is the lack of an immunologic confirmatory test in TRALI cases, aimed to detect the antibody-mediated nature of lung injury.^6^ From a clinical point of view, several pre-existing conditions (such as neurologic or valvular heart diseases) or new occurring diseases (such as infections, inhalation of gastric content during general anesthesia, amniotic fluid embolism) can cause respiratory distress during post-partum.^2^ The revision of clinical records allowed us to detect or to rule out frequent causes of ALI, like pneumonia, aspiration or sepsis 6. About amniotic fluid embolism (AFE), the reported incidence is very low, ranging from 1.9 to 6,1 cases per 100,000 maternities.^29^ Accordingly, among 22,344 deliveries occurred at our hospital in the same period of the study, no cases of AFE were observed. Finally, we could reasonably rule out TACO in our TRALI patients. TACO, usually, occurs in elderly people with poor cardiovascular function, often after FFP transfusion to reverse anticoagulation.^17^ Importantly, respiratory distress due to TACO rapidly improves with diuretic therapy.^17^ In contrast, in our patients with TRALI, the respiratory distress was not ameliorated by diuretic administration, suggesting that the pulmonary edema not be caused by cardiac dysfunctions or elevated systemic vascular resistance. Finally, we would emphasize that all patients included in the study were subjected to continuous monitoring of ECG, p02, blood pressure and breath rate in the delivery room. This procedure allowed the prompt detection of hypoxia and facilitated to define the timing of hypoxia onset in respect to transfusion.

## Conclusion

Our findings suggest that patients undergoing massive transfusion in post-partum are highly predisposed to develop TRALI: these observations can be particularly relevant in those countries where female donors with previous pregnancies or miscarriages as well as donors who had been previously transfused are still eligible for plasma donations. The strong association between pregnancy-related hypertensive disorders and TRALI predisposition deserves to be confirmed in a prospective evaluation. Moreover, future studies could definitely ascertain if therapies to treat hypertension and prevent pre-eclampsia also reduce the TRALI risk. In the meanwhile, the close observation of patients suffering from hypertensive disorders after transfusion should be recommended.

## Figures and Tables

**Table 1:** Univariate analysis of transfusion-related risk factors for TRALI. Significant results are in bold. RBC denotes red blood cell; PLT denote platelet; FFP denotes fresh frozen plasma; IQR denotes interquartile range; SD denotes standard deviation.

	TRALI/possible TRALI (n = 14)	No TRALI (n = 57)	*p*

RBC units Median number (IQR)	9 (3.75 – 13.25)	5 (3 – 6.50)	***0.008***
PLT units Median number (range)	0 (0 – 11)	0 (0–5)	***0.008***
FFP units Median number (IQR)	8 (2.25 – 15.50)	4 (0 – 8)	***0.034***
Total blood products from female donors Median number (IQR)	7 (3.5–7.5)	2 (1.7–4)	***0.047***
RBC units > 14 days Median number (IQR)	3 (0 – 7.25)	2 (0 – 3)	*0.175*
Storage time of RBC, days Mean+SD	15.79 ± 8.1	14.03 ± 6.3	*0.583*
Storage time of PLT, days Median number ( range )	4 (4–5)	1(1–5)	*0.350*
Number of patients receiving RBC+FFP+PLT n (%)	4 (29)	3 (5)	***0.025***

**Table 2:** Univariate analysis of patient-related risk factors for TRALI.

	TRALI/possible TRALI (n = 14)	No TRALI (n = 57)	*p*

Age, years Mean value ± SD	34,4 ± 5.8	34,4 ± 4.8	*0.940*
Smoke, n (%)	1 (7)	7 (12)	*1*
DIC, n (%)	6 (43)	21 (37)	*0.227*
Fibrinogen, mg/dlMean value ± SD	378,5 ± 117.1	406,7 ± 79,4	*0.235*
Hemoglobin, g/dl Mean value ± SD	10,9 ± 1,4	10,7 ± 2,1	*0.293*
Previous pregnancies Median number (IQR)	1 (1 – 3)	2 (1 – 3)	*0.231*
Parity, Median number (IQR)	0	0 (0–1)	*0.388*
Vaginal/Caesarean	3/11	18/39	*0.592*
Surgical intervention, n (%)	9 (64)	21 (36)	*0.077*
Pre-existing morbidities, n (%)	7 (50)	14 (24)	*0.099*
Non- hypertensive pregnancy- related co-morbidities, n (%)	2 (14)	5 (9)	*0.618*
Pregnancy-related Hypertensive disorders*, n (%)	5 (36)	3 (5)	***0.006***
Post-partum hospitalization, days Median number (IQR)	14 (10 – 291)	7 (5 – 12)	***<0.0001***
ICU admission, n (%)	5 (36)	5 (9)	***0.021***
Estimated blood losses, ml Median number (IQR)	2.000 (1.000–2.075)	1.850 (1.000–2.500)	*0.909*

* Pregnancy-related hypertensive disorders include three patients with pre-eclampsia and 2 patients with hypertension in the TRALI group and 1 patient with pre-eclampsia and 2 patients with hypertension in the no-TRALI group.

Significant results are in bold. ICU denotes intensive care unit; DIC denotes disseminated intravascular coagulation. Other abbreviations as in Table 1.

**Table 3:** Multivariate analysis of transfusion- and patient-related risk factors for TRALI. OR denotes Odds ratio, CI denotes Confidence Interval. Other abbreviations as in Table I. Significant results are in bold.

	OR	95% CI	*p*

RBC units	0.83	0.37 – 1.85	*0.652*
FFP units	1.33	0.71 – 2.50	*0.368*
PLT units	1.16	0.37 – 3.59	*0.790*
Pre-existing morbidities	0.23	0.13 – 4.27	*0.325*
Surgical intervention	1.96	0.15 – 25	*0.604*
Blood products from female donors	1.20	0.57 – 2.52	*0.626*
Pregnancy-related hypertensive disorders	27.7	1.27 – 604.3	***0.034***
